# Do implementation issues influence the effectiveness of medications? The case of nicotine replacement therapy and bupropion in UK Stop Smoking Services

**DOI:** 10.1186/1471-2458-9-28

**Published:** 2009-01-21

**Authors:** Andy McEwen, Robert West

**Affiliations:** 1 Cancer Research UK Health Behaviour Research Centre, University College London, London, WC1E 6BT, UK

## Abstract

**Background:**

Effective pharmacotherapies are available for smoking cessation but their efficacy is established through randomised controlled trials where the medication is supplied direct to subjects. In health care settings patient access to medicines is often less direct. The process for obtaining supplies of nicotine replacement therapy (NRT) is relatively easy for smokers attending National Health Service (NHS) Stop Smoking Services in the UK, whilst this is not necessarily the case for those wishing to using prescription only medicines (e.g. bupropion and varenicline). This study was a direct comparison of the short-term validated abstinence rates of NRT and bupropion in a clinical setting.

**Methods:**

Data were routinely collected from 2626 clients setting a quit date (82% of those registering) with two London NHS Stop Smoking Services that offered behavioural support combined with pharmacotherapy (NRT and bupropion).

**Results:**

Contrary to what would be expected from multiple randomised controlled trials, the CO-validated 3–4 week abstinence rate in clients using NRT was higher than for bupropion (42% versus 34%, p = .003). This difference persisted even when controlling for smoking characteristics, demographic variables and treatment variables 1.40 (95% CI = 1.08 – 1.83).

**Conclusion:**

Given that the level of behavioural support received by clients on each medication was identical, the most plausible explanation for the difference in effectiveness between NRT and bupropion perhaps lies with how clients of the Stop Smoking Services obtained their medications. Obtaining NRT was relatively easy for clients throughout the study period whilst this was not the case for bupropion. This study suggests that implementation issues and/or self-selection may influence the effectiveness of medications in health care, as opposed to research, settings.

## Background

The efficacy of pharmacotherapies is established through randomised controlled trials where the medication is supplied direct to subjects. In health care settings patient access to medicines is often less direct and this may influence compliance with, and effectiveness of, medications.

Both nicotine replacement therapy (NRT) and bupropion (Zyban) are of proven efficacy in helping smokers to quit. The most recent Cochrane review of multiple randomised controlled trials of NRT found that the overall odds ratio for long-term (6-month) abstinence with NRT, irrespective of additional support, compared to placebo was 1.77 (95% confidence intervals: 1.66 – 1.88) [1]. A Cochrane review of over thirty trials of bupropion established that the odds ratio for achieving abstinence from smoking for at least six months was 1.94 (95% confidence interval: 1.72 to 2.19) [2]. Analysis of the three head-to-head trials of bupropion and NRT included in the second of these reviews also revealed that bupropion is slightly more effective in helping smokers to achieve long term abstinence from smoking: the overall odds ratio was 1.34 (95% confidence interval: 0.71 to 2.56) [3-5].

A UK Government white paper in 1998 established the importance of the treatment of tobacco dependence and laid out a strategy for a NHS (National Health Service) Stop Smoking Service [6]. Funding to create and develop these stop smoking services was made available to all areas by 2000 and by the end of 2001 most were fully operational [7]. Bupropion became available on NHS prescription in June 2000; a supply of four to six weeks of NRT was obtainable via a voucher scheme operated by NHS Stop Smoking Services up until April 2001 when it too became available as an NHS reimbursable drug treatment for smoking cessation [8].

This paper details previously unreported prior medication use of clients attending NHS Stop Smoking Services and what medication clients' chose to use to aid their quit attempt. Our data allows us to report on usage of different NRT products, and of bupropion, and to consider the effectiveness of these medications in assisting clients achieve short term abstinence in a clinical as opposed to research setting. The data also allows for a direct comparison to be made between the effectiveness of these medications as the level of behavioural support received by clients on each medication type is identical.

There is evidence that providing free NRT to smokers can induce a large number to make a quit attempt [9]; and that making obtaining NRT easier (by switching the medication from prescription only to over the counter [OTC]) can result in increased use of NRT [10]. However, these large population-based cohort studies do not reflect what goes on in clinical practice and to our knowledge there are no studies that have considered how different routes of access to smoking cessation medications can influence their effectiveness.

## Methods

The research took place at two NHS Stop Smoking Services covering, in total, five outer London boroughs (Merton, Sutton & Wandsworth; Redbridge and Waltham Forest) with a combined population of about one million. These boroughs represent a diverse mix of inner city and suburban residential areas where the percentage of people of working age claiming key social benefits is 10% (Merton, Sutton & Wandsworth), 14% (Redbridge) and 18% (Waltham Forest); the average for London is 15% [11]. In both of these distinct services, the treatment regimen comprised six weekly 1–2 hour clinic (group) or community (one-to-one) support sessions over five or six weeks. This 'withdrawal-oriented treatment' [12] is recommended in smoking cessation guidelines [13] and combines behavioural support and pharmacotherapy (NRT or bupropion). This regimen is described in more detail elsewhere [14] but basically involves a pre-quit session to inform clients of what treatment involves, including deciding upon what medication they are going to use to aid their quit attempt. Subsequent sessions inform clients about the nature of the tobacco withdrawal syndrome and provide advice on how to manage the symptoms, ensure that clients have a sufficient supply of medication and are using it properly and provide encouragement designed to bolster motivation to remain abstinent.

All NHS stop smoking services collect demographic data on clients plus abstinence data for four weeks post-quit. The services reported in this paper also routinely collected additional data including: past quit attempts, prior medication use, indicated cautions and contraindications, choice of medication, side effects of medication use and dependence on smoking – Fagerström test for nicotine dependence (FTND) [15]. Data were collected as part of routine clinical practice and all clients gave written permission for the data to be used for research purposes. For service evaluations like this approval from Research Ethics Committees (REC) is not required [16]. Advice was sought on this from Wandsworth REC who concurred.

Baseline data were collected by questionnaire prior to or on the first visit, generally one or two weeks before the quit date. Abstinence data were then collected on the quit date and weekly for four weeks post-quitting. Treatment outcome was defined according to the UK department of Health service monitoring guidance [17] which is consistent with the Society for Research in Nicotine and Tobacco guidelines [18]. This allows for a two week 'grace period' before complete cessation is required; thus short term abstinence is defined as self-reported abstinence two weeks after the quit day for the final two weeks of attendance at the service, with a record of an expired-air CO reading of less than ten parts per million for these final two sessions. Only 1.7% (n = 77) of cases of self-reported abstinence were not verified by an expired-air CO reading of less than ten parts per million. These self-reported quitters who were not CO-validated, plus non-quitters (n = 291) and clients lost to follow up (n = 126) were all classed as non-abstinent. Data were entered on computer in anonymised form and analysed using SPSS version 13.0 [19].

### Analysis

Categorical and continuous data were analysed using chi-squared tests and t-tests respectively by abstinence status. A series of simple univariate logistic regression analyses were conducted to look at the relationship between CO-validated 3–4 week abstinence and gender, age, tobacco dependence (FTND), marital status, presence of other smokers in household, employment status, educational achievement, eligibility for free prescriptions, service, service type (clinic or community) and choice of medication (bupropion or NRT). A forward stepwise multiple logistic regression analysis was then conducted predicting short-term abstinence including those variables significant to p < .05. A forward stepwise multiple logistic regression analysis was also conducted predicting short-term abstinence comparing the different forms of NRT controlling for all the variables remaining in the previous forward stepwise regression.

Of the 3,200 smokers who registered with the services between January 2001 and December 2003, 2,626 (82%) set a quit date and it is on these that data are reported.

## Results

The vast majority (82%, 2034/2496) of clients attending the NHS Stop Smoking Services had attempted to stop smoking in the past; 27% of clients (698/2496) reported having made a single previous attempt and 54% (1336/2496) more than one attempt. The mean number of previous serious attempts to stop smoking was 2.2 (range 0–39, SD = 2.4). The median longest number of weeks abstinent for these previous quit attempts was 8 weeks (range 0–780). Over half (56%, 1430/2543) of clients who set a quit date were eligible for free prescriptions (e.g. were on low incomes, elderly or suffered from chronic health conditions); 44% (1113/2543) had to pay the then standard charge of £6.20 (approximately $12.40 or €7.85) per individual prescription item. Table 1 shows client characteristics of the study sample, which are typical of smokers attending for treatment in the UK [20,21], with a comparison for those choosing NRT and those choosing bupropion to aid their quit attempt.

**Table 1:** client characteristics

	Total sample^1 ^(n = 2,626)	NRT^2 ^(n = 1810)	Bupropion^2 ^(n = 388)
	% (n)/mean (sd)		

Sex: Female	59% (1536)	59% (1062)	57% (220)

Age*	45 (13.8)	46 (14.1)	44 (12.7)

Ethnicity: White*	85% (2183)	85% (1503)	91% (339)

Married/living with partner	51% (1299)	51% (897)	53% (197)

Education >16 years of age**	41% (1015)	41% (687)	55% (163)

In paid employment***	46 % (1172)	44% (767)	60% (222)

Age started smoking	16.7 (7.9)	16.7 (5.1)	17.3 (17.0)

No of cigarettes per day	21.4 (10.1)	21.4 (10.4)	22.2 (9.5)

Smoking hand-rolled	21% (517)	21% (366)	21% (78)

FTND	5.7 (2.2)	5.7 (2.2)	5.8 (2.1)

Use of medication for mental health problem***	13% (103)	17% (79)	4% (8)

Difference between NRT and bupropion significant to *p < .01; **p < .05 and *** p < .001

^1 ^Represents the number of smokers setting a quit date with the services within the study period

^2 ^Combined these represent the number of clients for whom medication use is recorded (n = 2238), where totals do not correspond it is because of missing values.

Sixty-three percent (1634/2579) of clients reported having used NRT before. Data on other previous medication use by clients was not available from the community setting of one service and so the following findings are from a subset of 1,947 clients. Only 10% (195/1879) of clients reported having used bupropion before. Prior use of the patch was reported by 47% (880/1885) of clients, 33% (626/1879) had tried gum, 12% (228/1876) the inhalator, 6% (103/1876) the sublingual tablet and 2% (44/1873) the nasal spray. Data on past use of the nicotine lozenge were only collected by the community treatment arm of one service; 4% (30/787) of clients reported having used it.

Three-percent (52/1907) of clients were contra-indicated for bupropion; 5% (91/1907) presented with a condition under which it should be prescribed with extreme caution and 12% (232/1904) with caution. At the end of their first consultation all 2626 clients were asked which medication they were going to use: 15% (388/2238) chose to use bupropion, 69% (1810/2238) NRT and for 17% (428/2238) their decision was unrecorded. Eighty-three percent (1175/1385) of clients who reported having used NRT before chose to use it again for their current quit attempt compared with 76% (605/779) of clients who had not used NRT previously (χ^2 ^= 17.8, p < .001). Similarly, 37% (59/159) of clients who had used bupropion before chose to use it for this quit attempt compared with 17% (233/1373) who had not used this medication before (χ^2 ^= 38.3, p < .001).

Nearly one quarter (222/989) of those in full time employment chose to use bupropion compared with 13% (151/1143) of those not in employment (i.e. unemployed, retired, students and carers) (χ^2 ^= 31.3, p <.001). Likewise a larger percentage of those not eligible for free prescriptions (23%, 215/930) chose bupropion compared with 13% (156/1202) of those who were on low incomes (χ^2 ^= 37.5, p <.001). There was also a difference in choice of medication according to ethnicity, 18% (339/1842) of white clients chose bupropion compared with 11% (33/301) of non-white clients (χ^2 ^= 9.98, p = 0.002). There were also differences in who chose to use bupropion according to which service clients attended (23%, 297/1309 [Service 1] v 10%, 91/889 [Service 2], χ^2 ^= 56.5, p < .001) and to whether they attended the clinic (group) or community (one-to-one) arm (31%, 222/729 v 11%, 166/1469 respectively, χ^2 ^= 122.9, p < .001). Data on the presence of "serious reactions" for bupropion were collected by the community (one-to-one) treatment arm of both services on the quit date: 6% (12/202) of clients reported their presence and were advised to discontinue the medication.

Overall the CO-validated 3–4 week abstinence rate was 41% (865/2129); for clients using bupropion it was 34% (129/377) and for those using NRT it was 42% (736/1752) (χ^2 ^= 7.81, p = .005). More dependent smokers, as measured by the FTND, were less likely to be abstinent over this period (t = 3.61, df = 2183, p < .001). There was no difference in short-term abstinence rates according to which service clients attended (p = .46); but CO-validated 3–4 week abstinence rates were higher for clients treated in clinics (i.e. group treatment) (43%, 388/911) than those treated in the community (i.e. individual treatment) (38%, 626/1638) (χ^2 ^= 4.67, p = .031).

Male clients were more likely than female clients to be abstinent at weeks 3 and 4 (42%, 445/1054 and 38%, 569/1488 respectively; χ^2 ^= 4.08, p = .043); as were older clients (t = -6.16, df = 2492, p = < .001). Short term abstinence rates also differed according to whether clients were married (44%, 557/1255), separated or divorced (35%, 152/431) or single (36%, 243/668) (χ^2 ^= 19.02, p < .0001). There were no differences in abstinence rates according to whether clients were eligible for free prescriptions or not (p = .06), whether they had made a prior quit attempt p = .06), whether clients had made one or more than one previous quit attempt (p = .14) or how long their previous quit attempt had lasted (p = .55).

Forty four percent (54/123) of clients reporting using at least 14 bupropion tablets (range 14–30) in the week prior to their quit date (the minimum recommended) were abstinent at 3–4 weeks compared with 32% (12/38) of those using below this amount (range 1–13); however this difference did not reach statistical significance (p = .26). Table 2 shows the results of a series of simple univariate logistic regression analyses conducted to look at the relationship between CO-validated 3–4 week abstinence and smoking characteristics and relevant demographic data.

**Table 2:** Simple regression analysis predicting short term abstinence from smoking characteristics and demographic data*

	Odds Ratio	P value
Gender (female/male)	1.18	.044

Age	1.02	<.001

Marital status (single, divorced or separated/married or with partner)	1.43	<.001

FTND	.93	<.001

Other smokers in household (no/yes)	.96	.78

Past serious quit attempt (no/yes)	1.22	.06

Employment (not in full time/in full time)	1.12	.19

Education (no qualifications/GCSE or above)	1.00	.98

Free prescriptions (not eligible/eligible)	.86	.06

Stop Smoking Service (1/2)	1.06	.46

Service type (community/clinic)	1.20	.031

Medication choice (bupropion/NRT)	1.39	.005

* This table reports on a number of separate simple univariate regressions to determine the independent effect of variables upon short term abstinence.

Table 3 shows the results of a forward stepwise multiple logistic regression of the items that reached significance of p < .05 in the simple univariate regressions, including choice of medication (bupropion/NRT). Use of NRT predicted greater chances of quitting compared with use of bupropion. The chances of achieving short term abstinence was also greater among older clients, male clients, clients in a stable relationship and those attending clinic services compared with community services. Greater levels of dependence as measured by the FTND predicted lower chances of achieving CO-validated 3–4 week abstinence.

**Table 3:** Forward stepwise regression predicting short term abstinence*

	Odds ratio	P value
Medication choice (bupropion/NRT)	1.40	.012

Gender (female/male)	1.30	.009

Age	1.02	<.001

Marital status (single, divorced or separated/married or with partner)	1.33	.003

FTND	.94	.006

Service type (community/clinic)	1.36	.012

* This table presents the output from a forward stepwise multiple regression where all the variables reaching significance in the univariate analyses (Table 2) were included in the model.

Table 4 shows the percentage choosing different NRT products amongst those clients making a quit attempt using NRT and the short term abstinence rates of those using NRT by NRT type. There were no significant differences in CO-validated 3–4 week abstinence rates according to which type of NRT clients chose to use to help them with their quit attempt (χ^2 ^= 4.25, p = .51).

**Table 4:** Choice of NRT medication by product and short-term abstinence rates by type of NRT used; % (n)

Product	All clients (n = 1810)	NRT type	3–4 week CO-validated abstinence (n = 502)
Inhalator	10.2 (185)	Inhalator	37.2 (67)

16 hour patch	19.4 (352)	Patch	43.0 (535)

24 hour patch	51.7 (936)		

2 mg gum	1.5 (27)	Gum	35.3 (30)

4 mg gum	3.3 (59)		

Nasal spray	2.5 (46)	Nasal spray	38.6 (17)

1 mg lozenge^1^	.1 (2)	Lozenge	42.7 (44)

2 mg lozenge^1^	1.3 (23)		

4 mg lozenge^1^	1.4 (25)		

Lozenge^2^	3.0 (55)		

Microtab	5.5 (99)	Microtab	44.3 (43)

^1 ^Service one only collected data on strength of lozenge. ^2 ^Second service lozenge data

Table 5 shows the results of a forward stepwise multiple logistic regression predicting short-term abstinence comparing the different forms of NRT controlling for all the variables in the previous forward stepwise multiple regression (see Table 3). None of the NRT types independently predicted short-term abstinence when controlling for other variables.

**Table 5:** Forward stepwise regression analysis between medication choice, smoking characteristics and demographic data and short term abstinence*

	Odds ratio	P value
Inhalator	-	.23

Patch	-	.32

Gum	-	.47

Nasal spray	-	.65

Lozenge	-	.93

Microtab	-	.60

Gender (male/female)	1.39	.003

Age	1.02	<.001

Marital status (single, divorced or separated/married or with partner)	1.33	.009

FTND	.95	.03

Service type (community/clinic)	1.46	.002

* This table presents the output from a forward stepwise multiple regression where variables reaching significance in the univariate analysis (Table 3) were included in the model alongside choice of NRT product. All of the NRT variables did not make it into the final model.

Data collected on amount of medication used after the quit date were not particularly useful because of differing requirements (e.g. one patch per day compared with 10–15 pieces of gum), but also because generally only abstinent clients continued to attend week on week. However, even among returning clients no use of medication was increasingly reported as the time since the quit date increased: 0.7% (8/1143) at week one post-quit, 1.9% (19/1002) at week two, 3.5% (31/892) at week three and 5.1% (45/878) at week four.

A sub-group of 822 clients, from the clinic arm of Service 1, responded to a baseline question about whether they were currently taking medication for a mental health problem; 12.5% (103/822) reported using such medication. Ninety one percent (79/87) chose to use NRT and 9% (8/89) bupropion. The overall short-term quit rate for clients currently taking medications for mental health problems was 34%; 37% for those using NRT and 25% for bupropion (χ^2 ^= 0.5, p = .41).

## Discussion

We might have expected bupropion to be more effective than NRT, as the clinical trials suggested, but we found the opposite. Also, bupropion was used more by clients who were white, in employment, not eligible for free prescriptions and who attended clinics as opposed to community advisers. Although some NRT products produced better short-term cessation rates than others these differences were not reflected in the logistic regression. Previous serious quit attempts, irrespective of whether they were the only prior quit attempt or how long they lasted, did not result in lower cessation rates.

Obtaining NRT was relatively easy for clients throughout the study period. In the first year, clients wanting to use NRT were given a voucher at their first appointment (one or two weeks prior to their quit date) for a free supply of their chosen product, redeemable at their local community pharmacy, which they could use on their quit date. In the second year when NRT was available on prescription clients were given a letter which recommended a GP prescription of their chosen product; both services reported that in most cases the client did not need to see the GP but only to pick up the prescription from the surgery. Whereas NRT is commenced on the quit date, bupropion has to be taken 8–14 days prior to the quit date, allowing time for a steady state concentration to be reached [22]. A letter was issued from the Stop Smoking Services requesting that the GP consider a prescription of bupropion, the client would then need to book and attend an appointment and start taking the medication at least a week prior to the quit date. Clearly where the first appointment was only one week prior to the quit date this would be impossible; even when such an appointment was two weeks prior to the quit date fulfilling this obligation would be a tall order. In many cases it is likely that bupropion was not taken 8–14 days prior to the quit date, a counter-intuitive regimen that may itself have contributed to sub-optimal adherence, and one can only surmise at the implications of this for the effectiveness of the medication. Additionally, bupropion was only recommended for prescription 'with behavioural support' [23] and the protocol for both services was for patients approaching GPs for a prescription to be directed to the Stop Smoking Service. The services would conduct an assessment of suitability for bupropion and then send the client back to the GP with a letter recommending bupropion. Continued prescriptions for bupropion would be dependent not only on abstinence, but on attendance at the Stop Smoking Service. It is plausible that smokers wishing to quit with the aid of bupropion alone were 'forced' into accepting behavioural support from their local NHS Stop Smoking Service. Indeed this was the intention of the licensing instructions, but it is unclear what affect the delay in quitting in order to obtain bupropion, and forced attendance at treatment sessions, had upon the dynamics of individual quit attempts.

It is unknown what effect the milieu of quit attempts has upon the chances of achieving abstinence. This is especially true of clients included in this study who were using bupropion, as they were doing so in an atmosphere where there was uncertainty over the safety of the drug. A series of newspaper articles published in February 2001 profiled a number of deaths in smokers using bupropion [24] and the story was picked up by other newspapers and by the BBC. The number of people receiving prescriptions of bupropion fell from 29% to 21.5% in the next two quarters [25], the public enthusiasm for bupropion had suddenly changed [26] and it is possible that this had some impact upon how bupropion was perceived and used by clients in this study.

Additionally, subjects enrolled in clinical trials are different from smokers attending clinical services [27]. At the very least, research subjects are normally expected to: smoke a minimum number of cigarettes per day (usually 10), be both physically and mentally healthy, be literate and be under the age of 75. The level of behavioural support provided to subjects is also often relatively intensive compared with routine clinical practice, and varies in intensity between different clinical trials. Therefore, less is known about the effectiveness of these medications in a 'real world' clinical setting. It is reassuring that a combination of behavioural support and use of NRT or bupropion was effective for clients reporting mental health problems, a group of patients usually excluded from clinical trials.

Clients who previously used NRT and bupropion were more likely to choose the same medication again for this current quit attempt. Interestingly, a serious quit attempt in the past did not result in lower cessation rates; neither did whether they had made one or more than one attempt or how long this previous attempt had lasted. Reported gum use was high for prior use but significantly lower for medication chosen for current quit attempt. This most probably reflects that previous NRT use involved OTC purchases.

As has been found previously, attendance at clinics improved client's chances of quitting successfully compared with attending community advisors for treatment. [14]. The client group included in this study was fairly typical of those attending NHS services [20,21]: there was a significant percentage not in full time employment, the majority were eligible for free prescriptions and mean FTND score was relatively high. The data are limited by the fact that this was a sample of relatively heavy smokers attending treatment to help them stop, but this sample is important in its own right. Data collected on medication usage proved not to be reliable and was unable to be meaningfully analysed; this should be a consideration for similar future studies. There was also a large number of 'missing values' for many items; this may be typical of data collected as part of routine clinical practice but should be addressed in future research, possibly through staff education. A further limitation of this paper is that it reports on end of treatment (four week) abstinence from smoking. However, the relapse rates for these clients after four weeks of abstinence would be expected to be similar to those described in a review of long term relapse curves [28] and we can deduce from this that the likely long term effect of treatment type is clinically important.

## Conclusion

The level of behavioural support received by clients on each medication was identical; therefore the most plausible explanation for the difference in effectiveness between NRT and bupropion appears to lie with the ease with which clients of the Stop Smoking Services obtained NRT as opposed to bupropion. The role that press reports on the safety of bupropion had upon the attitude of those clients taking it towards the medication and their quit attempt is unknown.

The labile nature of human motivation and the tendency towards relapse for those who are dependent upon tobacco suggests that, once engaged in a quit attempt, access to smoking cessation medications should be made as easy as possible. This is especially relevant now with the recent introduction of varenicline. Recent adverse press reports on the safety of varenicline [e.g. [29,30]] draw further parallels with bupropion and health professionals involved in smoking cessation may need to be prepared to answer queries from the public on such matters.

## Competing interests

AMc and RW have both received research and travel funding from, and undertaken consultancy for, manufacturers of smoking cessation medications (GlaxoSmithKline Consumer Healthcare, McNeil Healthcare (UK) Limited, Novartis Consumer Health and Pfizer Ltd). This study was part-funded by Cancer Research UK.

## Authors' contributions

AMc and RW were equally responsible for study design, data collection, data analysis and writing of paper.

## Pre-publication history

The pre-publication history for this paper can be accessed here:


